# Meet Me on the Other Side: Trans-Bilayer Modulation of a Model Voltage-Gated Ion Channel Activity by Membrane Electrostatics Asymmetry

**DOI:** 10.1371/journal.pone.0025276

**Published:** 2011-09-27

**Authors:** Loredana Mereuta, Alina Asandei, Tudor Luchian

**Affiliations:** Department of Physics, Laboratory of Molecular Biophysics and Medical Physics, Alexandru I. Cuza University, Iasi, Romania; Institut Curie, France

## Abstract

While it is accepted that biomembrane asymmetry is generated by proteins and phospholipids distribution, little is known about how electric changes manifested in a monolayer influence functional properties of proteins localized on the opposite leaflet. Herein we used single-molecule electrophysiology and investigated how asymmetric changes in the electrostatics of an artificial lipid membrane monolayer, generated oppositely from where alamethicin - a model voltage-gated ion channel - was added, altered peptide activity. We found that phlorizin, a membrane dipole potential lowering amphiphile, augmented alamethicin activity and transport features, whereas the opposite occurred with RH-421, which enhances the monolayer dipole potential. Further, the monolayer surface potential was decreased via adsorption of sodium dodecyl sulfate, and demonstrated that vectorial modification of it also affected the alamethicin activity in a predictive manner. A new paradigm is suggested according to which asymmetric changes in the monolayer dipole and surface potential extend their effects spatially by altering the intramembrane potential, whose gradient is sensed by distantly located peptides.

## Introduction

A key challenge faced by systems biology is to gain an understanding of the physical mechanisms that govern peptide adsorption, insertion and activity into lipid membranes. Among other factors, the membrane-water interfacial region was proven to play an important role in membrane association of proteins and peptides, due to the steep polarity gradient from apolar region near the hydrocarbon core of the membrane to highly polar, in the vicinity of the aqueous phase [1], [2]. In relation to this, it is well established that the membrane electrostatics has the potential of modulating manifestations of a wide selection of membrane proteins, including voltage-gated ion channels [3], enzymes [4], ligand-gated channels [5], antimicrobial peptides [6] and G-protein-coupled receptors [7]. In a broad description, the membrane electrostatics contains three major contributions: the transmembrane potential, stemming from a charge gradient across the membrane, the surface potential, generated by the net charge on the membrane surface, and the membrane dipole potential, whose origin lies in the oriented dipoles of bound water molecules, lipid headgroups, and lipid carbonyls located on the membrane lipid molecules [8]. The membrane dipole-potential whose magnitude is approximately 200 ÷ 300 mV [8], [9] and acts over a zone of roughly 1.0 nm, generates an electric field of approx. 2÷3×10^8^ V/m and plays important roles in cellular physiology, such as the translocation of hydrophobic ions through lipidic bilayers [8], it modulates the activity of phospholipase A2 [10], it alters the extent of the membrane fusion [11], as well as the insertion and channel forming activity and single channel properties of peptides and proteins (*vide infra*). In addition, changes in the bilayer shape which accompany the partitioning of water soluble peptides into lipid bilayers contribute to the free energy of insertion, and involves mechanical contributions stemming from its elastic properties (thickness, intrinsic lipid curvature, and the elastic compression and bending moduli) [12]. The bilayer-induced allosteric regulation of protein function has been remarkably well described explained in studies involving various antimicrobial peptides useful for ion channels representation [13]–[16].

One of the most remarkable structural features of living cells biomembranes is their asymmetry with respect to the lipid compositions in the two monolayers [17]. This is currently perceived as a significant determinant of membrane properties through differences in fluidity, alteration of membrane protein environment, or changes in transmembrane electric field, of vital importance for membrane properties and functions [18], [19]. Lipid asymmetry was proven to alter the electric field across the membrane and contribute with a nonzero potential difference between the two opposing monolayers even in the absence of ion charge gradients across the membrane [20]–[22]. This in return is crucial for lipid flip-flop rate, membrane-based signaling dynamics, the modulation of voltage-gated proteins activity, enzymes, transport through ionic channels, binding and translocation of various small molecules and peptides across cell membranes [23]–[26].

To more effectively decipher mechanisms regarding the regulation of membrane protein function by electrical and mechanical properties of lipid membranes, it is usually useful to focus on particular frameworks able to simplify the *in-vivo* structural and functional features of biomembranes, and yet provide relevant, biological insight. Alamethicin is a 20-residue peptide from the fungus *Trichoderma viride*, which forms voltage-activated oligomers with well-defined conductance levels. Because these channels are relatively simple in structure and function, they constitute suitable model systems for understanding lipid membranes-peptide interactions and ion channel properties [27]. An intriguing property of alamethicin is its exponential voltage-dependent pore formation propensity, and when applied to the cis side of a membrane, only negative, trans applied potentials, lead to monomers insertion into the membrane and channel activation [27]–[29]. In the absence of voltage, membrane adsorbed alamethicin monomers adopt a partially N-terminal inserted interfacial orientation with the C-terminal part anchored to the interface by a number of peptide/lipid hydrogen-bonds from the side-chains of Glu-18 and Gln-19, and the terminal hydroxyl of Phl-20 to the phosphate, glyceryl and acyl oxygens of the lipid and water in the interfacial region [29]. Within a widely accepted model, the voltage-dependent insertion of cis-added alamethicin is caused by the interaction between the α-helical dipole of alamethicin and the transmembrane potential, leading to its insertion into the membrane where it undergoes further oligomerization.

Previous results have documented extensively that by manipulating controllably the sign of change and the magnitude of the interfacial dipole field by amphiphiles like phloretin, phlorizin, RH-421 and 6-ketocholestanol, added on both sides of the membrane or *on the side of peptide addition only*, it is possible to modulate the extent of the membrane penetration and transport properties mediated by alamethicin [30], gramicidin [31], [32], surfactin [33], syringomycin E [34], melittin and magainin 2 [35], analogues of the HP(2–20) antimicrobial peptide [36], the mitochondrial amphipathic signal sequence p25 [37], and the human immunodeficiency virus protease inhibitor saquinavir [38].

However, very little is known about how changes in the overall transmembrane potential profile caused by asymmetric alteration of the dipole and surface membrane potential of a trans membrane monolayer, extend spatially and manifest functional roles in modulating the kinetic and transport features of cis-added, model ion channel-forming peptides.

In this work we sought to understand the effects of asymmetry in the electrostatic features of a reconstituted lipid membrane, on dynamic properties of alamethicin. We used amphiphiles like phlorizin, styrylpyridinium dye RH-421 and sodium dodecyl sulfate (SDS), that preferentially partition into a single membrane leaflet, added vectorially to the membrane opposite to the side of alamethicin insertion, to controllably alter the dipole and surface potential, and provide a quantitative evaluation of the effects of asymmetric bilayer electric potentials on peptide function.

Our work strongly suggests that the dipole and surface potential asymmetry between the cis and trans leaflets of a reconstituted membrane acts as a transbilayer driving force strongly modulating alamethicin activity and ion transport properties. Additionally, we demonstrated that electric and mechanic modulatory effects induced by the anionic detergent SDS on alamethicin activity are un-coupled, whereby the canceling out of the electric contribution induced by SDS through symmetric addition on both sides of the membrane, left its mechanical effect on peptide activity still active. Data presented here lend support to the paradigm according to which asymmetric changes in the monolayer dipole and surface potential may play additional functional roles in regulating membrane protein function, and extend their effects spatially by altering the intramembrane potential, whose gradient is sensed by distantly located peptides.

## Materials and Methods

Electrophysiology on alamethicin oligomers was performed on the folded bilayer membranes system, obtained with Montal-Mueller technique, as we described previously [16], [30]. In short, a lipid bilayer was formed from L- α-phosphatidylcholine (Sigma-Aldrich, Germany) in pentane, on an aperture of 100 µm diameter in the Teflon septum that had been pretreated with 10% (v/v) hexadecane (Sigma-Aldrich, Germany) in highly purified n-pentane (Sigma-Aldrich, Germany), which separates the cis and trans bilayer chambers of 1 mL in volume. Both chambers contained 0.5 M KCl (for the experiments with RH-421 and phlorizin) or 0.1 M KCl (for the experiments with SDS) salt solutions buffered at a pH value of 6.5 in 10 mM HEPES buffer (Fluka, Germany). Experiments with SDS were carried out in 0.1 M KCl in order to ensure the proper solubilization of the detergent molecules, since higher salt concentrations more effectively screens out the electrostatic interactions among them and facilitate their hydrophobic clustering. All experiments were performed at a room temperature of 25°C. Alamethicin monomers (Sigma-Aldrich, Germany) were added from a stock solution made in ethanol, only to the cis chamber which was connected to the ground. Mechanical stirring was initiated in this chamber for several minutes to ensure proper concentration homogenization. Spontaneous peptide insertion was usually obtained under stirring at holding potentials of −80÷−100 mV.

When employed, styrylpyridinium dye RH-421 (Sigma-Aldrich, Germany) or phlorizin (Fluka, Germany) were added to the trans side of the membrane, from stock solutions made in ethanol, kept under dark at 4°C. The anionic detergent sodium dodecyl sulfate (Sigma-Aldrich, Germany) was added from a stock solution of 5 mM made in distilled water. Most importantly, before adding any amphiphile to the bilayer chamber containing the dissolved peptide, we waited long enough (tens of minutes under stirring), to allow peptide molecules to reach the stationary state with respect to their partitioning to the lipid membrane. Therefore, we sought to avoid a mis-interpretation of a subsequent alteration in alamethicin activity following any agent addition, which might have occurred simply as a result of re-homogenization of peptide monomers within the membrane. Furthermore, to make sure that under our working conditions the time-lag between adding alamethicin and amphiphiles was long enough to avoid this problem, control experiments were run during which we monitored whether the activity of alamethicin oligomers changed upon simply stirring of the solution in the trans side. Only in those instances where the activity of alamethicin remained largely unchanged following such ‘ghost-stirring’ in the trans chamber, did we proceed with amphiphiles addition.

The electrical connection between the bilayer chamber and the amplifier was made via Ag/AgCl electrodes. Currents from the bilayer chamber, which was housed in a Faraday cage (Warner Instruments, USA) and mechanically isolated with a vibration-free platform (BenchMate 2210, Warner Instruments, USA), were detected and amplified with an EPC 8 patch-clamp amplifier (Heka, Germany) set to the voltage-clamp mode. Data acquisition of the amplified electrical signals was performed with a NI 6251, 16-bit acquisition board (National Instruments, USA) at a sampling frequency of 20 kHz within the LabVIEW 8.20 environment. Data were than fed into a PC-compatible computer for further numerical analysis and graphing, done mainly with the help of the Origin 6.0 (OriginLab Corporation, USA) and pClamp 6.03 (Axon Instruments, USA) software.

To construct the I–V diagrams of currents mediated by various substates of the alamethicin oligomer in the absence or presence of various amphiphiles, we quantified electrical currents corresponding to such substates from original current recordings, without resorting to amplitude histogram analysis, due to the thermal noise which in certain instances precluded the separation between the first substate of alamethicin from its closed state. Usually, more than 20 readings of the electrical current corresponding to a certain open state at a given holding potential were employed.

## Results and Discussion

**Figure 1:**
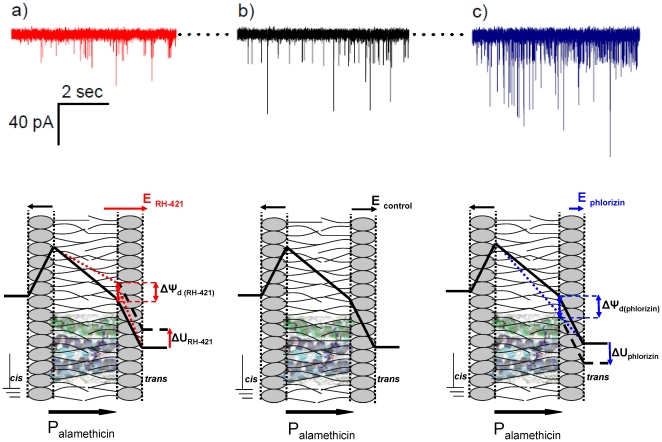
Representative current recordings which illustrate the alamethicin activity in lipid membranes under control conditions (no amphiphile added, panel b), and presence on the trans side of the membrane of either RH 421 [8 µM] (panel a), or phlorizin [500 µM] (panel c). The applied potential was −60 mV. The closed state of alamethicin oligomers is denoted by the dotted line, and downward spikes designate the electrical current mediated by alamethicin pores in their various conductive state. Beneath it is shown an over-simplified geometric view of changes ensued on the overall membrane potential profile, by the increase (panel a, dotted line) or decrease (panel c, dotted line) of the trans-monolayer dipole potential (Δψ_d_) of the *trans* lipid monolayer only, as compared to control conditions (panels a, b, and c, solid line). When the dipolar electric field - initially similar for both the cis and trans monolayers (E_control_) - is altered in the trans monolayer only, as a consequence of RH-421 (E_RH-421_ > E_control_) or phlorizin (E_phlorizin _< E_control_) adsorption, a corresponding change in the intramembrane potential across the hydrophobic region of the membrane will follow. As a result, the net potential difference sensed by the cis-side adsorbed alamethicin over the hydrophobic region while crossing the membrane, will equal that seen under control conditions at a given trans-applied potential, from which a given value must be subtracted (denoted by ΔU_RH-421_; panel a, dashed line) or added to (denoted by ΔU_phlorizin_; panel c, dashed line). Based on the presented simplified geometric and electric considerations, this value (ΔU) should match the change brought about by either amphiphile adsorption on the trans-monolayer dipole potential (Δψ_d_). The solid arrows assigned to P_alamethicin_ indicate the orientation of alamethicin monomers dipole moment, while in the transmembrane orientation.

In the first part of this study we undertook a comparative, quantitative analysis of the effects of RH-421 and phlorizin on alamethicin oligomer activity and its electric conductance, when both dipole-potential modifiers amphiphiles were added asymmetrically, opposite to the membrane side of alamethicin addition. As shown in Fig. 1, panel a, the trans side addition of 8 µM RH-421, known to increase the dipole potential of the monolayer where it inserts to, leads to a reduction in alamethicin activity as compared to control conditions (no amphiphile added) (Fig. 1, panel b). As stated before, alamethicin was added to the grounded, cis side of the membrane.

To probe further the effect of asymmetrically added dipolar compounds modifiers on alamethicin activity, phlorizin, an amphiphile known to decrease the monolayer dipole potential, was injected on the trans side of the membrane, at an aqueous concentration of 500 µM. Fig. 1, panel c, illustrates the augmentation of the cis side added alamethicin oligomerization, caused by phlorizin interaction with the trans monolayer.

To explain this, we neglected the contribution of small, negative surface potentials which are present even in the case of zwitterionic phospholipids membranes [39], and took into consideration only the dipole and transmembrane potential electrostatic profile of the membrane, shown diagrammatically in Fig. 1, beneath current traces. On both monolayers, hydrated lipid headgroups behave as an array of inward-pointing dipoles giving rise to an interfacial dipolar structure viewed for our purpose as a continuum phase, whose electric field vector points outwardly and generates the interfacial dipole potential, positive towards the membrane interior. In the absence of surface charges at the hydrophobic-interfacial layer interface, the normal component of the dielectric displacement is continuous, and so is the electric potential. Therefore, when the trans side of the bilayer membrane is subjected to a negative potential with respect to the grounded cis side, the lumped transmembrane potential profile varies across the membrane as shown in Fig. 1, panel b.

By the virtue of the same physical arguments, when the dipole potential profile is altered in either monolayer, a corresponding change in the intramembrane potential will ensue. In this framework, we posit that trans-side injection of RH-421 leads to a reduction of the intramembrane potential difference across the hydrophobic region of the membrane (Fig. 1, panel a). As a result, the net electric field sensed by the cis side adsorbed alamethicin monomers, at a given negative holding potential established in the trans compartment, will decrease. The immediate effect would be an increased energy barrier along the alamethicin transition path towards the membrane-inserted state, whereby the magnitude of the cis-to-trans oriented external electric field determines the propensity of the initially interfacially oriented peptide helix dipole, to cross the membrane to the inserted position. Similarly, to explain the augmenting effect on alamethicin activity at a constant applied holding potential by the trans-adsorbed phlorizin, we propose as a major factor the net increase of the electric field within the membrane core caused by the asymmetric decrease of the dipole potential in the trans-monolayer (Fig. 1, panel c).

**Figure 2:**
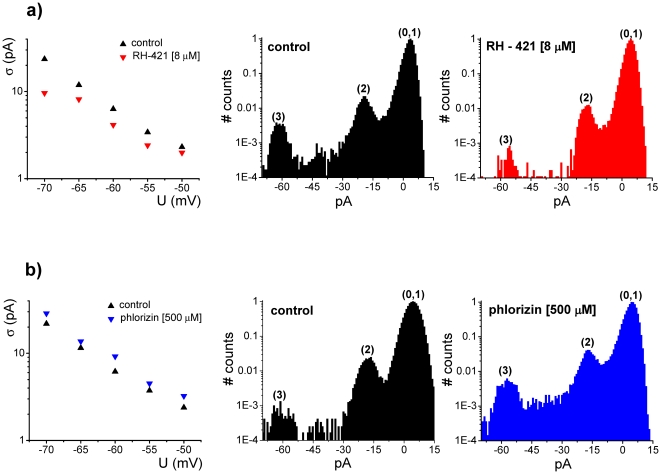
Quantitative description of the modulatory effect by exerted RH-421 (panel a) and phlorizin (panel b) on alamethicin activity, added asymmetrically to the trans side of a membrane, via estimations made on the standard deviation (denoted by σ) of the electrical current mediated by alamethicin oligomers at different holding potentials, before and after amphiphile adsorption, as well as the probability of appearance of high-conducting substates on alamethicin aggregates, inferred from the normalized amplitude histogram of current fluctuations seen in the absence (control) and presence of adsorbed amphiphiles at −60 mV. Due to the inherent thermal noise, the first substate (1) on the alamethicin oligomer is poorly discernable from its closed state (0) on the amplitude histogram. Therefore, the area of convoluted Gaussian component denoted by (0, 1) represents the probability of appearance of either ‘closed’ or first substate on alamethicin's reversible oligomerization. Areas assigned to peaks denoted by ‘2’ and ‘3’ provide a quantitative view of the probabilities to which the second, and respectively third conductive substates appear during alamethicin's reversible oligomerization.

In Fig. 2 we present a quantitative view of the modulatory effect exerted on alamethicin activity by the two amphihiles (RH-421 and phlorizin), added asymmetrically to the trans side of a membrane, through estimations made on the standard deviation of the electrical current fluctuations, as well as the probability of appearance of high-conducting substates on alamethicin oligomers.

Numerical estimations revealed that at an applied potential of −60 mV, trans-injection of RH-421 reduced the probability of appearance of higher conductance states with ∼54%, whereas phlorizin augmented with it with ∼74%.

**Figure 3:**
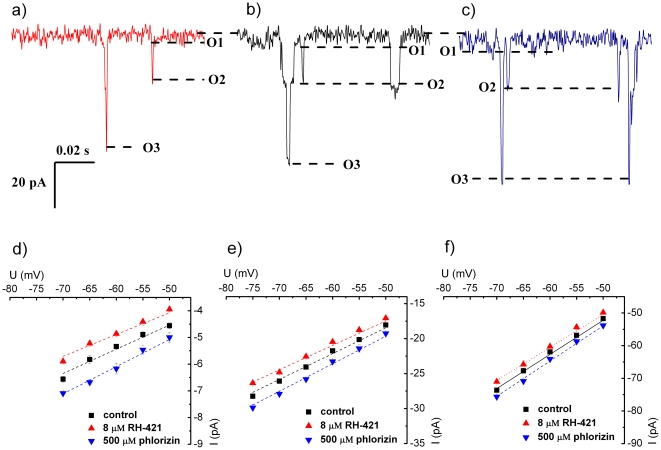
Magnified view of traces recorded at −60 mV showing the current amplitudes mediated by the first (O_1_), second (O_2_) and third (O_3_) sub-conductance states of the alamethicin oligomer under control conditions (no amphiphile added, panel b), and presence on the trans side of the membrane of either 8 µM RH 421 (panel a), or 500 µM phlorizin (panel c). Shown below are representative I–V diagrams illustrating the ion current at various transmembrane potentials, mediated by the first (O_1_) (panel d), second (O_2_) (panel e) and third (O_3_) (panel f) alamethicin substates in the absence (control) and presence of either RH 421 or phlorizin.

RH-421 and phlorizin not only entailed changes in the membrane activity of alamethicin oligomers, but also caused alterations of transport properties of alamethicin distinct conductive substates. Single-channel recordings on alamethicin oligomers made at various holding potentials, in the absence and presence of RH-421 and phlorizin, revealed a consistent alteration of currents mediated by the first three substates of the alamethicin oligomer when either amphiphile got adsorbed to the trans monolayer. As shown in Fig. 3, phlorizin led to an increase of ionic charge transfer through alamethicin, whereas RH-421 caused the opposite.

From the linear fitting of I-V diagrams shown in Fig. 3, we calculated the conductance values of the first, second and third substates of the alamethicin oligomers during control experiments, which equal 0.09±10^−3^ nS, 0.37±10^−3^ nS and 1.04±7×10^−3^ nS, respectively (mean ± s.e.m). From similar evaluations, we inferred that the trans-added RH-421 (8 µM) reduced the first, second and third substates conductance values to 0.08±10^−3^ nS, 0.35±10^−3^ nS and 1.01±5×10^−3^ nS (mean ± s.e.m), whereas phlorizin (500 µM) enhanced them to 0.1±5×10^−4^ nS, 0.4±2×10^−3^ nS and 1.08±5×10^−3^ nS (mean ± s.e.m), respectively. The simplest physical explanation we propose for such changes relies on the Goldman-Hodgkin-Katz formalism [40] in conjunction with conclusions inferred previously regarding the effects of RH-421 and phlorizin on the transmembrane potential profile. That is, adsorption of either RH-421 or phlorizin to the trans monolayer under a clamped negative transmembrane potential, and subsequent increase or decrease in the trans-monolayer dipole potential, leads to an overall change of the potential profile across the membrane. In the framework of the Goldman-Hodgkin-Katz formalism and with relevance to our data, it is worth recalling that the net value of the electric current is not only proportional to the difference of exponential potential values maintained across the membrane, but also inversely proportional to the exponential of the integral value of the potential profile, calculated across the permeating pathway. In a simplified view (e.g., we neglect the contribution of polarization charges induced at the membrane/aqueous pore boundary caused by diffusing ions through alamethicin, etc.) we propose that the trans-added, RH-421 adsorption leads to an alteration of the membrane potential profile, which in the end is equivalent to an increase in the energy barrier anions and cations must surpass in order to permeate through alamethicin. This in turn leads to a decrease in the net electric current recorded at various holding potentials [9]. By following a similar reasoning, it can be argued that the opposite happens when phlorizin interacts with the trans monolayer.

As pointed out before (see Fig. 1), simple electrostatics and geometric reasoning supports the observation that the degree of trans-monolayer dipole potential alteration induced by either RH-421 or phlorizin, equals the change one may have to impose on the transmembrane potential *in the absence of amphiphile adsorption*, to arrive at a similar electric potential profile over the hydrophobic region of the bilayer, and consequently comparable changes in alamethicin activity. Based on this, we further used the computed changes in the membrane activity of alamethicin oligomers in the absence and presence of RH-421 [8 µM] and phlorizin [500 µM], to make quantitative estimations of absolute values to which the trans dipole potential changed, as a result of their membrane asymmetric adsorption.

**Figure 4:**
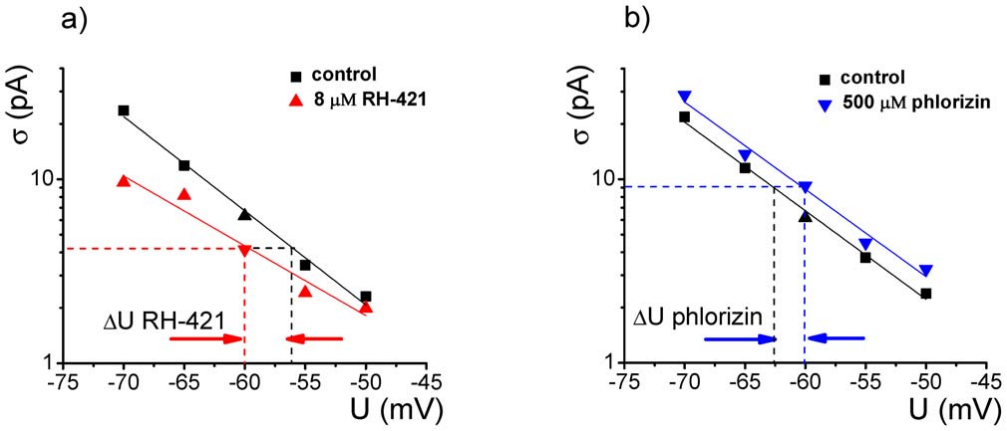
Illustrative diagrams showing changes in the membrane activity of alamethicin oligomers in the absence and presence of trans-added RH-421 (panel a) and phlorizin (panel b), quantified through estimations made on the standard deviation (σ) of the electrical current mediated by alamethicin at different holding potentials, used to make quantitative estimations of absolute values to which the trans dipole potential changes as a result of amphiphile asymmetric adsorption. Based on the rationale presented in the text, we posit that the dipole potential increase brought about by the RH-421 [8 µM] adsorption on the trans-monolayer at any given transmembrane potential is equivalent to a decrease with ∼3.7±2 mV (mean ± s.e.m) of the applied holding potential (denoted by ΔU_RH-421_) and no amphiphile added (control conditions), in order to arrive at a similar alamethicin activity. Similarly, and by comparison with control experiments, the trans-monolayer dipole potential decrease mediated by phlorizin adsorption [500 µM] increases the alamethicin activity to an extent as seen under control experiments, whereby the applied transmembrane potential would increase with roughly 2.8±0.8 mV (mean ± s.e.m) (denoted by ΔU_phlorizin_).

As we illustrate in Fig. 4, and with the simplifying assumption that such amphiphile chemical potential is relatively un-influenced by the applied transmembrane potential [41], [42], we posit that the dipole potential increase brought about by the RH-421 adsorption on the trans-monolayer at any given transmembrane potential is equivalent to a decrease with ΔU_RH-421_∼4 mV of the applied holding potential and no amphiphile added (control conditions), in order to arrive at a similar alamethicin activity. Similarly, at any given holding potential and by comparison with control experiments, the trans-monolayer dipole potential-decrease mediated by phlorizin adsorption increases the alamethicin activity to an extent as seen under control experiments, whereby the applied transmembrane potential would increase with roughly ΔU_phlorizin_∼3 mV.

Further, we calculated the extent to which the altered transmembrane electric field caused by trans-side injection of RH-421 or phlorizin alters the relative energetics between interfacial and transmembrane orientation of alamethicin. We first proposed the following the simplifying assumptions according to which: (i) in its interfacial orientation, alamethicin lies mostly parallel to the membrane surface, and the transmembrane electric field created by a negative trans-potential induces a transition to a perpendicular orientation of alamethicin, spanning the entire hydrophobic region of the membrane (ii) upon trans-adsorption of either amphiphile, the change brought on the spontaneous curvature of the trans-monolayer, which is among the known factors that modulate alamethicin insertion [43] does not influence appreciably the alamethicin insertion. By multiplying the alamethicin dipole moment (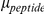
∼75D) by the net change in the electric field (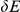) sensed in its inserted orientation over the hydrophobic thickness of a typical phospholipid membrane (h∼3 nm), in the presence of either RH-421 (
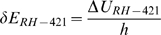
) or phlorizin (
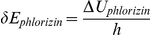
) at their given concentration (*vide supra*), we conclude that the energy difference between the interfacial and transmembrane orientation of the peptide (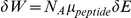; N_A_ is Avogadro's constant) are 0.19 kJ mol^−1^ (RH-421) and 0.14 kJ mol^−1^ (phlorizin). As suggested above, these numbers are relative indicators of energetic contributions for alamethicin reorientation and incorporation into the membrane, provided by trans-side adsorption of either RH-421 [8 µM] or phlorizin [500 µM].

To investigate in further details how asymmetric alteration of membrane electrostatics on one monolayer couples with alamethicin activity, when the peptide inserts into the membrane from the opposite side, we next used the sodium dodecyl sulfate (SDS) amphiphile. Among others, SDS is widely used as solubilizing agents of biological membranes. In addition, results from previous seminal work endows such detergents with exquisite membrane-active roles, able to modulate membrane protein function via changes brought about in the energetic cost of bilayer deformations associated with various protein and ion channels functioning [44], [45].

Due to its charged moiety, SDS exhibits a rather slow membrane flip-flop rate at room temperature (minutes to hours) [46]. This ensures that during the time scale of our experiments when SDS was added asymmetrically to one membrane monolayer, no equilibration occurs between leaflets. Membrane adsorption of SDS into a zwitterionic lipid membrane is favored by the hydrophobic interactions of the hydrocarbon chains of the lipids, and produces a negative surface charge due to the negatively charged head groups which impede, via electrostatic repulsion, the further insertion of SDS molecules [46].

**Figure 5:**
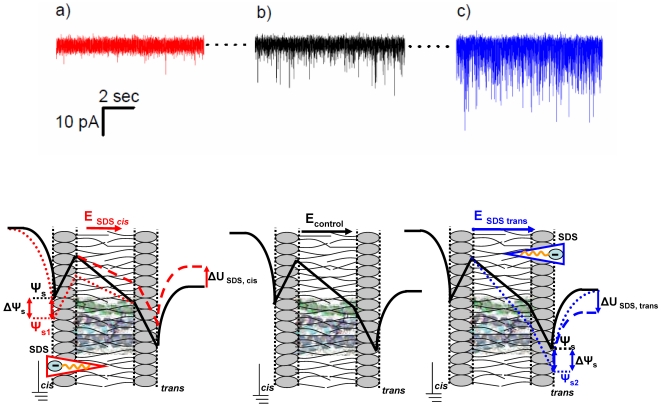
Representative current recordings mediated by alamethicin in lipid membranes under control conditions (panel b), and presence of 25 µM SDS on either the trans (panel c) or cis side (panel a) of the membrane. The closed state of alamethicin is denoted by the dotted line, whereas downward spikes represent electrical current through alamethicin oligomers at an applied potential of −55 mV. As it was used before (*vide supra*, Fig. 1), we display schematically beneath a simplified, geometric view of changes ensued on the membrane potential profile by the change of the membrane surface potential initiated by SDS adsorption on the cis (panel a, dotted line) or trans side (panel c, dotted line) of the membrane, as compared to control conditions (panels a, b, and c, solid line). The membrane surface potential in the control state (ψ_s_) decreases as a result of either cis (ψ_s1_) or trans-side (ψ_s2_) adsorption of SDS, whereas the membrane dipole potential remains un-affected. As a result, a change in the intramembrane electric field (E) across the hydrophobic region of the membrane will follow, as shown. The net potential difference sensed by the cis-side adsorbed alamethicin over the hydrophobic region of the membrane, will equal that seen under control conditions at a given trans-applied potential, from which a given value must be subtracted (ΔU_SDS, cis_; panel a, dashed line) or added to (ΔU_SDS, trans_; panel c, dashed line). These values (ΔU_SDS_) should match the change caused by either SDS adsorption on the surface potential of either monolayer (ΔΨ_s_).

In Fig. 5 we present representative traces which illustrate the antagonistic, strong modulatory effect exerted by 25 µM SDS on alamethicin activity and transport features, when the amphiphile is injected on either side of the membrane.

**Figure 6:**
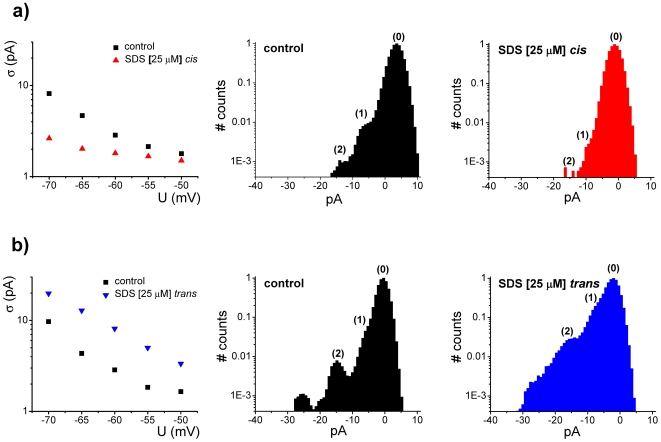
Quantitative description of the modulatory effect exerted on alamethicin activity by SDS [25 µM] added on the cis (panel a) or trans-side (panel b) of the membrane, via estimations made on the standard deviation (denoted by σ) of the electrical current through alamethicin oligomers at different holding potentials, before and after SDS adsorption, as well as the probability of appearance of various conducting substates on alamethicin aggregates, inferred from the normalized amplitude histogram of current fluctuations seen in the absence (control) and presence of adsorbed amphiphiles at −55 mV. Areas assigned to peaks denoted by ‘1’ and ‘2’ provide a quantitative view of the probabilities to which the first, and respectively second conductive substates appear during alamethicin's reversible oligomerization (*vide supra*, Fig. 2), before and after SDS injection.

In quantitative terms and as already described, such changes seen in alamethicin activity and ion transport were further described by the electric noisiness of alamethicin oligomers (standard deviation of alamethicin-mediated currents) and probability of appearance of highly-conductive substates with SDS injected asymmetrically on either side of the membrane (Fig. 6).

By the virtue of similar physical reasoning as presented above, we propose that the asymmetric presence of a negative surface charge on either trans or cis side of the membrane, generated by SDS partitioning, leads to a negative drop in the surface potential of the corresponding monolayer. Consequently, the intramembrane potential profile will be altered, so that trans-adsorption of SDS will generate a steeper potential gradient across the membrane hydrophobic core – which energetically favors the insertion of the cis-added alamethicin – whereas the opposite occurs when SDS adsorbs on the cis side of the membrane.

**Figure 7:**
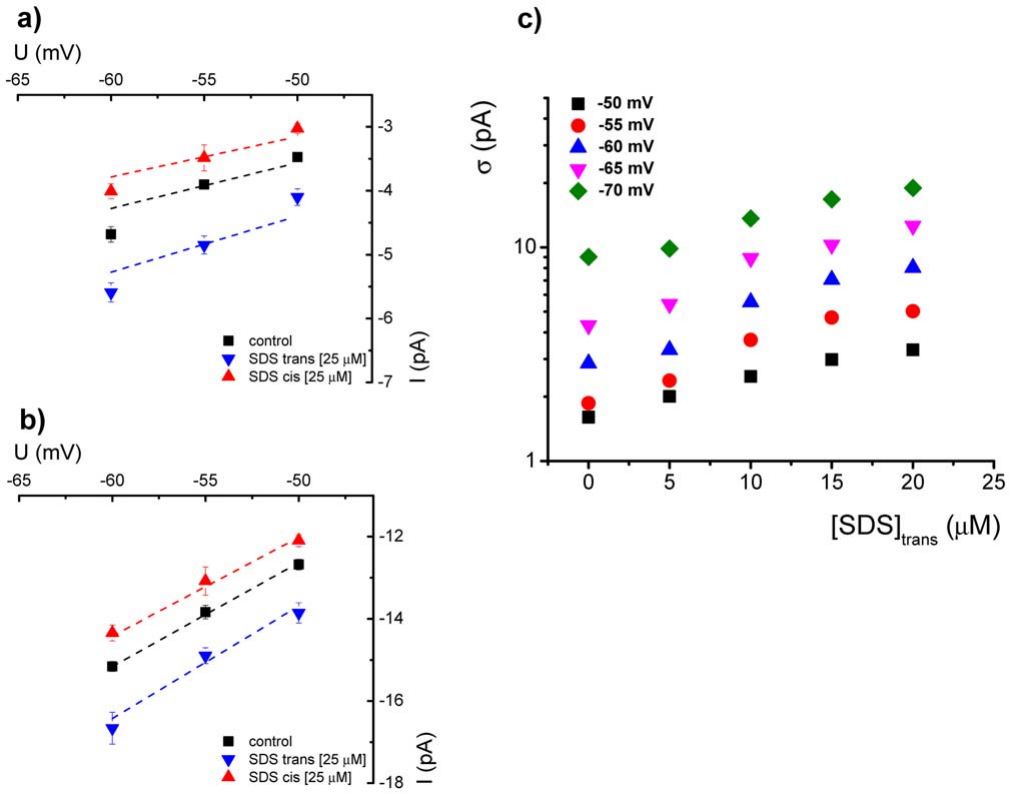
Representative I–V diagrams showing the ion current values at various transmembrane potentials, mediated by the first (panel a) and second (panel b) sub-conductance states of alamethicin in the absence (control) and presence of SDS injected asymmetrically on either cis or trans side of the membrane. In panel c are shown SDS concentration- and voltage-dependent changes imposed in alamethicin activity, as quantified by the standard deviation (σ) of the electrical current through alamethicin oligomers, when SDS was injected only on the trans side of the membrane. It is apparent that at any given holding potential, the alamethicin activity quantified by the logarithm of current fluctuation standard deviation, increases linearly with the amphiphile concentration. This is in accordance with the physical fact that the membrane surface potential of the trans monolayer increases linearly as a function of the SDS bulk concentration within low concentration values of the amphiphile; in turn, it is predicted a linear dependence of potential difference over the hydrophobic core of the membrane vs. the bulk, trans-injected SDS concentration, and consequently an exponential increase in alamethicin activity.

The elevated conductance values (0.09±3×10^−3^ nS for the first substate and 0.27±2×10^−3^ nS for the second substate), and respectively reduced conductance values of a single alamethicin oligomer in its various conductive substates (0.06±2×10^−3^ nS for the first substate and 0.24±10^−3^ nS for the second substate), measured in the presence of either trans- or cis-adsorbed SDS, as compared to control conditions (0.07±2×10^−3^ nS for the first substate and 0.25±5×10^−4^ nS for the second substate) (mean ± s.e.m) (Fig. 7, panels a and b), reflect in our view an augmented or decreased net electrical force acting on migration ions through the alamethicin pore, and the physical explanation can be tackled within the Goldman-Hodgkin-Katz formalism (*vide supra*).

Within a similar formalism as described above, we calculated the absolute values of the surface potential changes of the trans and cis monolayer, induced by asymmetric SDS injection on either sub-phase of the membrane, by the changes one may have to impose on the transmembrane potential *in the absence of SDS adsorption*, to arrive at an electric potential profile across the hydrophobic region of the bilayer able to give rise to similar changes in the alamethicin activity. We posit that at a concentration of 25 µM, the trans-added SDS augments the transmembrane potential difference, and implicitly that seen over the hydrophobic core of the membrane, with a value equal to the change brought on the trans-monolayer surface potential, ΔU_SDS, trans_∼10 mV, and it decreases it with the value ΔU_SDS, cis_∼8 mV, when SDS is injected on the cis sub-phase only. In the limit of low SDS bulk concentration, these numbers are in good qualitative agreement with previously found values of the membrane surface potential calculated for POPC membranes, from experiments with isothermal titration calorimetry [46]. The small discrepancy seen with respect to the extent to which SDS alters the surface potential of either monolayer is poorly explained at the moment.

In terms of transition energy alteration of alamethicin from the interfacial to inserted state, as it occurs with SDS adsorbed in either trans or cis monolayer, via intermembrane changes of the electric potential determined by decrease values of the membrane surface potential, within the framework described above we calculated a 0.5 kJ mol^−1^ relative change caused by trans-adsorbed SDS and a 0.4 kJ mol^−1^ relative change by its cis-adsorption.

The relationship between the surface charge density caused by SDS adsorption and the surface potential can be inferred by using the Grahame equation, which relates the surface charge density of SDS to the surface potential, and the Langmuir adsorption isotherm of SDS to a surface with saturable binding sites, leading to the Stern equation [40], [46]. The most straightforward outcome of this formalism, relevant to our work, is that membrane surface potential of the membrane monolayer where SDS partitions to, increases linearly as a function of the SDS bulk concentration within low concentration values of the amphiphile ([SDS]<250 µM) [46]. We therefore expected a linear dependence of membrane surface potential vs. bulk, trans-injected SDS concentration and consequently predicted an *exponential* increase in alamethicin activity, caused by resulting equidistant changes ensuing on the intramembrane potential profile [47].

Gratifyingly, our data fitted nicely this prediction. Shown in Fig. 7, panel c, are SDS concentration- and voltage-dependent changes in alamethicin activity, when SDS was injected only on the trans side of the membrane with equal amounts. It is visible that at any given holding potential, the alamethicin activity quantified by the logarithm of current fluctuation standard deviation, increases linearly with the amphiphile concentration.

To provide further experimental support of our mechanistic interpretation of SDS effect on alamethicin activity, another prediction we tested referred to the reversible change in alamethicin activity following amphiphile addition in a pre-defined order during the same experiment (first to the trans side, than to the cis side, or reverse). We expected that the change in the intramembrane potential profile caused by asymmetric addition of SDS, and implicitly alamethicin activity, would be reversed when a similar amount of detergent is present on the opposite side of the membrane.

**Figure 8:**
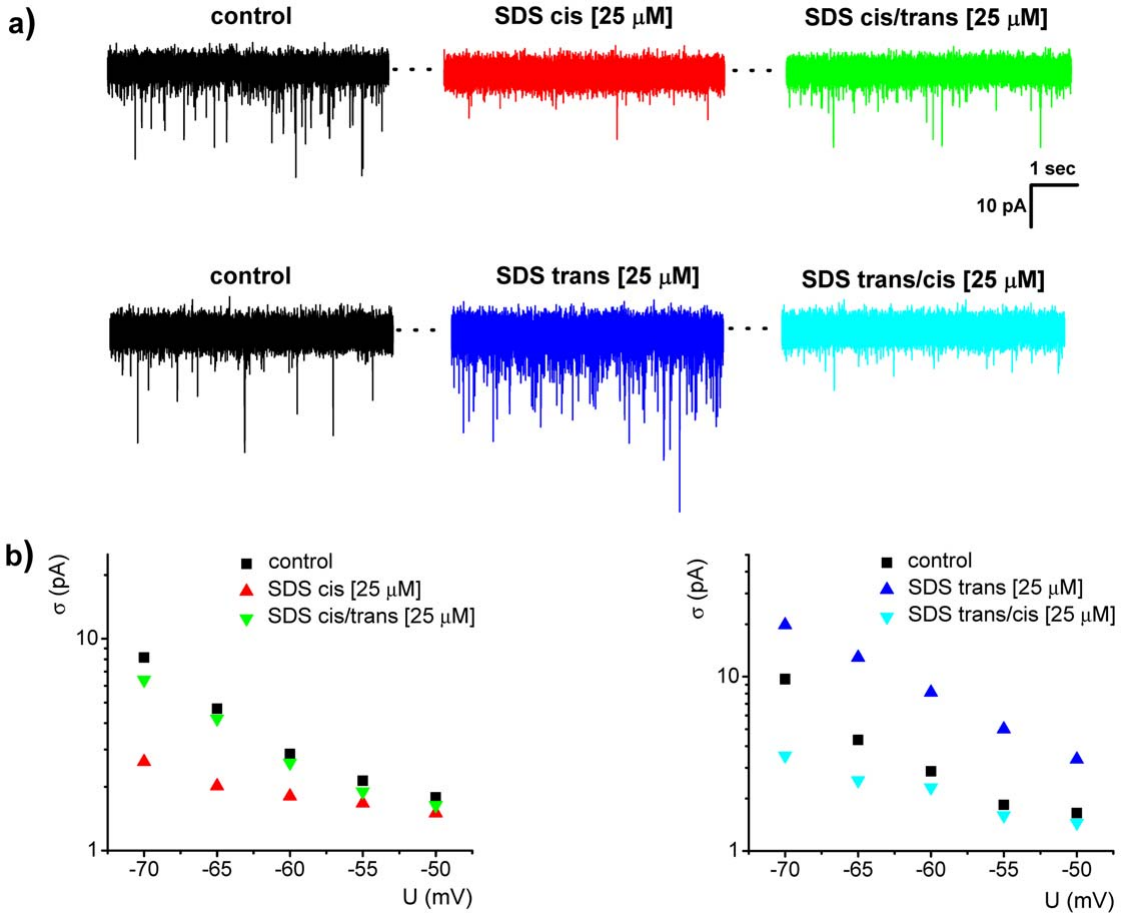
Representative current traces measured at −55 mV demonstrating the reversible change in alamethicin activity with respect to control condition following SDS addition in a pre-defined order during the same experiment, first to the cis side only (cis), and than to the trans side (cis/trans), or reverse (i.e., SDS added the trans side only (trans), and than to the cis side (trans/cis)) (panel a). This is suggestive of the paradigm according to which changes in the intramembrane potential profile caused by asymmetric addition of SDS on either cis or trans side, are partially reversed when a similar amount of detergent is present on the opposite side of the membrane. In panel (b) we present a quantitative analysis of this phenomenon, through estimating the standard deviation (σ) of the electrical current mediated by alamethicin oligomers at various holding potentials, under experimental conditions described above (see also text).

Data shown in Fig. 8 fully support this assertion. It is seen that the decrease in alamethicin activity caused by 25 µM SDS injection to the cis side of the membrane - with ∼22% at −55 mV as compared to the control state (Fig. 8, panel a; SDS cis) - is partially recovered to ∼89% of the initial activity, when a similar amount of SDS is added to the trans side of the membrane (Fig. 8, panel a; SDS cis/trans). When the experiment proceeded in the reverse order, the trans-injection of 25 µM SDS caused an increase with ∼166% of alamethicin activity as compared to the control state (Fig. 8, panel a; SDS trans), and the cis-addition of SDS at the same concentration reversed the activity to only 87%, as compared to the control state (Fig. 8, panel a; SDS trans/cis). In Fig. 8, panel b, we present a quantitative analysis of this phenomenon as it is observed at various applied potentials, through estimations made on the standard deviation (σ) of the electrical current mediated by alamethicin oligomers, under experimental conditions detailed above.

While the observed reversible changes in alamethicin activity reflects that the alteration of the intramembrane potential gradient caused by asymmetric addition of SDS is partially reversed when a similar amount of detergent is present on both sides of the membrane, the question still remains regarding the absence of full recovery of alamethicin activity following symmetric addition of SDS at a similar concentration. In addition, we performed similar evaluations whereby changes in the conductance of the first conductive state of the alamethicin oligomer were monitored during the same experiments, and the partial recovery of the alamethicin oligomer conductance in its first conductive state was observed (data not shown).

Due to the fact that all experiments were performed well below the CMC formation of SDS, a putative mechanism of action in which alamethicin is sequestrated by SDS micelles or other aggregates and therefore unavailable for interaction with the lipid bilayers, as it was proposed in alternative systems involving fluorinated amphiphiles and α-hemolysin proteins [48], is unlikely. To rationalize our data, it should be reminded that upon their insertion, SDS molecules impose a mechanical strain on the bilayer (i.e., they increase in spontaneous curvature of monolayer where they partition to) [49]. Previous data have established that DOPE lipids, which are prone to form inverted hexagonal phases and favor negative spontaneous curvature, shift the single-channel probability distribution of alamethicin oligomers towards higher conductance substates [50]. Equally interesting, in phosphatidylethanolamine and phosphatidylcholine binary lipid bilayers, increasing the mole fraction of the former precludes alamethicin binding to the lipids, and at the same time favors the oligomerization of membrane residing alamethicin monomers [51].

Based on this, it is conceivable that the membrane activity of alamethicin oligomers formed once aqueous alamethicin in the aqueous phase is in equilibrium with peptide in the membrane, would decrease as the spontaneous curvature of the bilayer is made more positive. This mechanism would then explain the residual smaller activity of alamethicin, with respect to the control case, under conditions of symmetrical partition of SDS amphiphiles (Fig. 8).

### Conclusions

In the view of recent literature, a great deal of effort is being devoted to studying the regulation mechanisms of membrane protein function by various amphiphiles, at concentrations that are prone to affect the physical properties of lipid membrane. One of the facets of this objective is to better understand the importance of membrane asymmetry, usually maintained by lipids, in cellular signalling and evolution of major diseases. Due to the fact that the large majority of pharmaceuticals are amphiphiles, it stems natural for pharmaceutical development to invest extensive knowledge into studying the effects of amphiphiles on membrane protein function. The mechanisms of membrane asymmetry and its disruption either by lipid themselves or various amphiphiles are thus beginning to unravel, and help to elucidate how important biological functions are crucially influenced by the membrane asymmetry.

In this article, we explored at the single-molecule level the role of the induced asymmetry in the membrane dipole- and surface-potential of an artificial bilayer on the intramembrane potential gradient, for the activity and transport properties of a model voltage-gated ion channel, alamethicin. We used amphipathic agents that insert into either leaflets of a planar lipid membrane and controllably alter the membrane dipole potential (RH-421 and phlorizin) or its surface potential (SDS), and to evaluated how altering the potential profiles of the trans-monolayer, opposite to that where alamethicin binds (the cis monolayer), affects formation of alamethicin oligomers and ion transport through them.

Our results indicate that the binding of the cis-added peptide to the membrane is greatly influenced by the sign of change of the dipole potential in the trans-monolayer, whereby phlorizin, a dipole-potential lowering agent, augments alamethicin activity, whereas the opposite happens with RH-421, a membrane dipole potential enhancing amphiphile. In a similar fashion, the ion conductance of the oligomeric alamethicin in its various substates is increased, and respectively decreased by the injection of phlorizin or RH-421 on the trans sub-phase. We attributed these changes to the overall change in the intramembrane potential profile whose gradient across the membrane is intimately linked to asymmetric changes in the membrane dipole potential, and is capable of coupling over the lipid membrane thickness, via electrostatic interactions, with the alamethicin peptide. The trans-monolayer potential profile effect upon alamethicin functioning was probed further, when the surface potential profile was independently altered via vectorial insertion of SDS. Our results confirm that either cis-only or trans-only monolayer modification of the membrane surface potential modulates at will and in a predictive manner the membrane insertion and transport properties of alamethicin, and this can be also accommodated within the intermembrane potential profile and its dependence upon asymmetric changes of the membrane surface potential formalism. In addition, we demonstrated the utility of alamethicin not only in probing, but also quantifying absolute membrane dipole- and surface-potential changes induced by these amphiphiles on an artificial lipid membrane. The presented approach is suggestive of an alternative molecular tool for quantitative explorations of the asymmetric, bilayer potential profile-mediated influence of peptide and protein activity.
